# Renal neutrophil gelatinase associated lipocalin expression in lipopolysaccharide-induced acute kidney injury in the rat

**DOI:** 10.1186/1471-2369-13-25

**Published:** 2012-06-27

**Authors:** Mei Han, Ying Li, Maodong Liu, Yingmin Li, Bin Cong

**Affiliations:** 1 Department of Nephropathy, The Third Hospital of Hebei Medical University, Shijiazhuang, 050051, China; 2 Department of Forensic Medicine, Hebei Medical University, Shijiazhuang, 050051, China

**Keywords:** Neutrophil gelatinase associated lipocalin, lipopolysaccharide, acute kidney injury, tumor necrosis factor α

## Abstract

**Background:**

Neutrophil gelatinase associated lipocalin (NGAL) is a highly predictive biomarker of acute kidney injury. To understand the role of NGAL in renal injury during sepsis, we investigated the temporal changes and biological sources of NGAL in a rat model of acute kidney injury, and explored the relationship between renal inflammation, humoral NGAL and NGAL expression during endotoxemia.

**Methods:**

To induce acute renal injury, rats were treated with lipopolysaccharide (LPS, 3.5 mg/kg, ip), and the location of NGAL mRNA was evaluated by in situ hybridization. Quantitative RT-PCR was also used to determine the dynamic changes in NGAL, tumor necrosis factor α (TNFα) and interleukin (IL)-6 mRNA expression 1, 3, 6, 12, and 24 hours following LPS treatment. The correlation among NGAL, TNFα and IL-6 was analyzed. Urinary and plasma NGAL (u/pNGAL) levels were measured, and the relationship between humoral NGAL and NGAL expression in the kidney was investigated.

**Results:**

Renal function was affected 3–12 hours after LPS. NGAL mRNA was significantly upregulated in tubular epithelia at the same time (*P* < 0.001). The course of NGAL mRNA upregulation occurred in parallel with renal damage. There was a transient increase in TNFα and IL-6 mRNA levels within 3 hours following LPS administration, and a strong correlation between TNFα and NGAL mRNA (r = 0.995, *P* <0.001) but not with IL-6 mRNA. Both pNGAL and uNGAL levels were markedly increased compared with those in the control group (*P* < 0.001); however, only uNGAL levels were correlated with NGAL mRNA (r = 0.850, *P* <0.001).

**Conclusions:**

NGAL upregulation is sensitive to LPS-induced renal TNFα increase and injury, which are observed in the tubular epithelia. Urinary NGAL levels accurately reflect changes in NGAL in the kidney.

## Background

Acute kidney injury (AKI; or acute renal failure) is an important issue for patients during critical care, with sepsis being the most common trigger for AKI in the intensive care unit (ICU) [1-3]. Because of the lack of sensitive and specific biomarkers for indicating renal cell injury, the mortality rates for septic AKI have remained high [1,4]. Recently, genomic, transcriptomic, and proteomic techniques have identified neutrophil gelatinase associated lipocalin (NGAL) as an early marker of AKI [5,6]. NGAL has been investigated in a range of different clinical settings, such as contrast-induced nephropathy, AKI after cardiac surgery or kidney transplantation and AKI in the critical care setting. Overall, the sensitivity for NGAL to predict AKI is 0.815 (95 % confidence interval, 0.732-0.892) and it is a promising biomarker for AKI, similar to troponin for acute myocardial infarction [7].

However, the use of NGAL in detecting or predicting the outcomes of patients with septic AKI is complex and depends on the clinical setting. In previous research of critically ill patients [8,9], both serum NGAL (sNGAL) and plasma NGAL (pNGAL) levels have the potential to act as early biomarkers for AKI. While both of them are highly sensitive, they have not been found to be very specific predictors of septic AKI. Shapiro et al. assessed the diagnostic accuracy of pNGAL in predicting AKI, wherein it was revealed that pNGAL concentrations of >150 ng/mL were 96 % sensitive but only 51 % specific for patients with suspected septic AKI[9]. Martensson et al. observed that pNGAL is elevated in patients with systemic inflammatory response syndrome, severe sepsis, and septic shock, and it should be used with caution as a marker of AKI in ICU patients with septic shock [10]. In contrast, studies have shown that urinary NGAL (uNGAL) is more useful for predicting AKI, as uNGAL levels are not elevated in septic patients without AKI. Understanding the role of NGAL in impaired renal tissues may help clarify this issue.

Furthermore, a biomarker should temporally reflect the pathophysiology initiated by a stimulus leading to injury so as to alert the clinician to a potentially reversible stage of the illness. In the case of septic AKI, the relationship between NGAL and early inflammation mechanisms in the kidney will ought to be clearly established. However, the mechanisms of septic AKI remain undefined. Increasing evidence suggests that intrarenal inflammatory mediators play an important role in the pathogenesis of kidney injury[11-14]. Specially, tumor necrosis factor α (TNFα) is a pivotal proinflammatory mediator and interleukin (IL)-6 is an accessory factor [15,16]. However, it remains unknown whether NGAL expression is related to changes in renal IL-6 and TNFα during the early stage of septic AKI.

Based on the available evidence, the use of NGAL as a marker of septic AKI is promising but requires further investigation. In particular, the relationship between NGAL expression, humoral NGAL and renal inflammation requires additional clarification. However, few studies have specifically investigated the expression of NGAL in renal tissues during sepsis. Therefore, the purpose of this study was to investigate the pattern and localization of renal NGAL expression, and to explore the relationship between renal inflammation mediators, such as TNFα and IL-6, and humoral NGAL in a rat model of lipopolysaccharide (LPS)-induced kidney injury.

## Methods

### Animals

Male Sprague–Dawley rats (Animal Laboratory of Hebei Medical University), 200-220 g, were used in this study. The rats were maintained for a 3-day period before the experimental procedure under a 12-h light/dark cycle at a constant temperature (23 ± 2°C), with free access to food and tap water. All animal experiments were in compliance with the guidelines stated by the Institutional Animal Care and Use Committee.

### Experimental groups

Endotoxemia and AKI were induced in the rats by injecting them intraperitoneally (i.p.) with LPS (*Escherichia coli* 0111: B4, Sigma-Aldrich). The LPS was dissolved in a concentration of 1 mg/ml in normal saline. Since the response to LPS is strongly dependent on ambient temperature [17], the environmental temperature was maintained at 24°C.

The rats were randomly assigned into six groups (n = 8 rats/group). Group 1, the control group, was treated with isometric sterile saline. Groups 2 to 6 were treated with LPS (3.5 mg/kg body weight). Differences between Groups 2 to 6 reflected the different time points following LPS administration at which sampling was conducted (1, 3, 6, 12 and 24 h). Urine was gathered via metabolic cages and the supernatant was obtained and collected. The animals were then anaesthetized with sodium pentobarbital (60 mg/kg i.p.), and 2 ml of blood was obtained by cardiac puncture and processed to collect plasma and serum samples, which were frozen at −80°C for subsequent use [18]. The kidneys were also harvested. Half of the kidney was fixed in paraformaldehyde and processed for hematoxylin and eosin (H&E) staining and in situ hybridization, and the other half was snap-frozen in liquid nitrogen and stored at −80°C for subsequent biochemical analysis.

### Evaluating renal function

Renal function, in the form of serum creatinine levels, was evaluated in the saline- and LPS-treated animals using a colorimetric assay (Creatinine Assay Kit; Biosino Bio-Technology). The serum creatinine levels were evaluated by measuring the change in absorbance over 40 seconds in the experimental samples relative to the standards.

### Histological studies

Histopathology was conducted on the kidney samples to determine the time-course of renal micro-morphological injury in the LPS-induced endotoxemia rats. Each sample of kidney (one quarter from both the control and experimental groups) was fixed in 4 % paraformaldehyde, dehydrated in graded ethanol and embedded in paraffin as previously described [19]. Each paraffin block was processed into 5-μm-thick slices that were H&E-stained. A portion (~1 mm^3^) of renal cortex from each rat was fixed in 2.5 % glutaraldehyde diluted in 0.066 M phosphate buffer (pH 7.4) for 24 hours. The samples were then dehydrated in a graded ethanol series and embedded in Epon 812 resin at 60°C for 48 hours. Thin sections (50 nm) were then double-stained with uranyl acetate and lead citrate, and were observed and photographed with a transmission electron microscope operated at 80 kV. The epithelial layer was examined and photographed with a transmission electron microscope (Hitachi, H-7500, TEM) at a magnification of 5000×.

### In situ hybridization

In situ hybridization was performed on 5-μm sections using an acetylcholine receptor in situ hybridization Kit (BioChain Institute, Hayward, CA) with some modifications to the instructions provided by the manufacturers. The following probes were used for whole-mount in situ hybridization: 5’-GCCTGGCAGCGAATGCGGTCCAGAAA GAAAGACAA-3’; 5’-AGGGGCCAGGGCGTGCACTACTGGATCAGAACATT-3’, 5’-GACTACGACCAGTTTGCCATGGTATTTTTCCAGGA-3’. Dewaxing, rehydration, and antigen retrieval were performed as previously described [20]. The sections were then incubated in diethyl pyrocarbonate (0.1 % DEPC)-treated PBS and fixed in 4 % paraformaldehyde in PBS for 10 minutes. After being rinsed twice with PBS, the slides were prehybridized with ready-to-use prehybridization solution (20 μl/slice) for 3 hours at 40°C. The DIG-labeled probe (0.15 μg/μl) was diluted to 20 ng/μl in ready-to-use hybridization buffer and applied to the prehybridized tissues. The sections then were incubated at 40°C for 16 hours. Posthybridization, the sections were washed and incubated for 2 hours in AP-conjugated anti-DIG antibodies (1:100, diluted in PBS). After the staining was developed, the reaction was stopped by incubating the slides in 10 mM Tris buffer (pH 8.0, 1 mM EDTA). Finally, the slides were rinsed in distilled H_2_O, counterstained with sappan wood, dehydrated with alcohol, transparented with xylene, and then sealed to take images with a dissecting microscope (Olympus, cx31).

### RNA extraction and quantitative reverse-transcription polymerase chain reaction (qRT-PCR)

RNA was extracted from the kidney samples using a single-step RNA isolation method [21] with the Total Quick RNA kit (D9108B; TaKaRa, Dalian, China). The purified RNA (500 ng) was quantified and reverse-transcribed using the PrimeScript RT reagent kit (DRR037A; TaKaRa, Dalian, China), according to the manufacturer’s instructions, yielding 20 μl of first-strand cDNA. The qRT-PCR experiments were measured on an ABI Prism 7500 RT-PCR machine (Applied Biosystems, Carlsbad, CA, USA). The different mRNA expression levels were then calculated and expressed as the number of copies per reaction. All experiments were performed in triplicate. The fold change in mRNA levels of each gene was calculated using the ▵ ▵CT method. The mRNA levels were normalized by using GAPDH as a housekeeping gene, and they were compared with the control group. The authenticity and size of the PCR products were confirmed by 2 % agarose gel electrophoresis imprinting. The primers used for amplification are listed in Table 1.

**Table 1:** Primers used for qRT-PCR

Gene	Sense	Anti-sense
TNFα	TACTCCCAGGTTCTCTTCAAGG	GGAGGTTGACTTTCTCCTGGTA
IL-6	TCCTTTGAACTCTACAAGGACC	GTATCCACCATTATGCCCAGCC
NGAL	GATGTTGTTATCCTTGAGGCCC	CACTGACTACGACCAGTTTGCC
GAPDH	GGCATGGACTGTGGTCATGA	TTCACCACCATGGA GAAGGC

Abbreviations: NGAL, neutrophil gelatinase associated lipocalin; TNFα, tumor necrosis factor α; IL-6, interleukin 6; GAPDH as a housekeeping gene.

### Plasma and urine NGAL concentrations

Plasma and urine samples were obtained from the rats at 1, 3, 6, 12, and 24 hours after the LPS (or saline) treatment, and the concentration of NGAL was determined using a commercially available ELISA kit (CSB-E09409r, Cusabio Biotech, China), in accordance with the manufacturer’s instructions. Briefly, microtiter plates pre-coated with a goat polyclonal antibody raised against rat NGAL were incubated in blocking buffer containing 1 % BSA, and then coated with 100 μL samples of either plasma, urine or NGAL standards (ranging from 1–200 ng/mL). The plates were then incubated with a horseradish peroxidase conjugated, affinity purified rabbit anti-goat IgG antibody, and a TMB substrate was added for color development. The plates were then read within 30 minutes (450 nm) with a Benchmark Plus microplate reader (Bio-Tek Instruments Inc., Winooski, VT, USA). All measurements were performed in triplicate.

### Statistical analysis

All values are expressed as mean ± SEM. The data were normally distributed, as determined using a Kolmogorov-Smirnov test. Comparisons among groups were conducted with one-way ANOVA. When the F value was significant (*P* < 0.05), the Student–Newman–Keuls Test and least significant difference procedure were performed to test for differences between the means. We used simple regression analysis after log transformation to evaluate whether the values were correlated. A *P* value of less than 0.05 was considered to be significant.

## Results

### LPS-induced AKI

**Figure 1:**
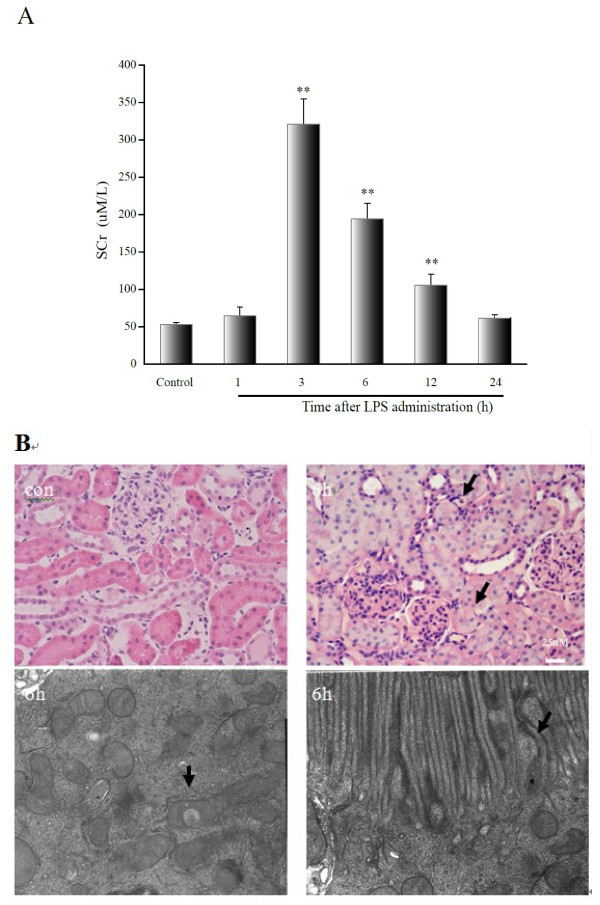
**SCr and renal histological injury in rats subjected to LPS at different time points.A**, SCr levels are shown, with the mean values/group obtained by colorimetric assay (uM/L). There was a significant increase in SCr levels in rats at 3, 6 and 12 hours. **B**, Renal histology under light microscopy and TEM at 6 hours following LPS treatment (H&E, 200×; TEM, 5000×) are shown. The arrows indicate intracellular edema, inflammatory cell infiltration, microvilli in disarray, or injury to the mitochondrial outer membrane. Abbreviations: SCr = serum creatinine. **P < 0.001, relative to the control group.

Rats treated with LPS showed significantly elevated levels of serum creatinine, which were significantly increased from 3 to 12 hours after LPS administration compared with those in the control group (*P* < 0.001, Figure 1A).

Histopathology was also performed to determine the time-course of renal damage following LPS treatment. The kidneys from the control group showed normal glomeruli and tubules. In contrast, the kidneys of the LPS-treated rats were characterized by transient renal damage. Under a light microscope, there were no intrinsic lesions within the glomeruli, but lesions consisting of epithelial swelling were observed in the 3–12-hour groups. Further, intracellular edema was also detected and was more severe in the straight segment of the proximal tubules in the LPS-treated rats. Ultrapathology analysis further demonstrated the presence of intracellular edema, coupled with disarranged microvilli, and/or injury or destruction of the mitochondrial outer membrane in the proximal epithelia (Figure 1B).

On the basis of changes to serum creatinine levels and renal histology, we were able to confirm LPS-induced AKI, and thus confirm that we had established a suitable animal model.

### Upregulation of NGAL gene expression in the tubular epithelia

**Figure 2:**
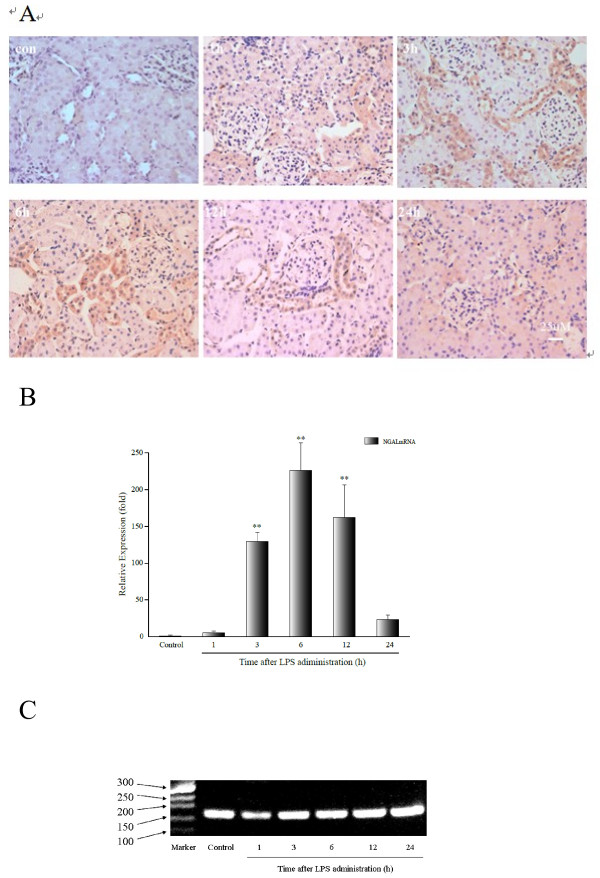
**Expression of NGAL mRNA in the LPS-treated rats.A**, In situ hybridization signal time-course. A strong hybridization signal was observed in the cortical tubular epithelia in samples in the 3-, 6- and 12-hour LPS groups compared with a weak signal in the 1- and 24-hour LPS groups, and there was no signal in the controls. **B**, Relative expression of NGAL mRNA (relative to the controls) is shown. **C**, Agarose gel electrophoresis imprinting expresses the intensity of NGAL mRNA in the LPS-treated rats. There was a 129-fold increase in NGAL mRNA expression at 3 hours after LPS treatment, rising to 226-fold at 6 hours and then it decreased to 162-fold at 12 hours. **P < 0.001, relative to the control group.

Using in situ hybridization analysis, we observed weak NGAL mRNA expression in the distal tubular epithelia and medullary collecting ducts of the control kidneys; however, NGAL mRNA expression was not detected by qRT-PCR. In contrast, 24.3-49.6 % of the tubular cells, including the proximal epithelia, were NGAL mRNA positive in the kidneys of Groups 2 to 5 (1, 3, 6 and 12 hours post-LPS treatment, respectively), whereas NGAL mRNA expression was significantly decreased in the kidneys from Group 6 (24 hours post-LPS treatment) (Figure 2A). This finding was also confirmed by qRT-PCR, where LPS-treatment induced the upregulation of renal NGAL mRNA from 3 to 12 hours following treatment compared with controls (*P* < 0.001). More specifically, at its peak, the expression of NGAL mRNA increased by 260-fold (Group 4, 6 hours post LPS), and decreased to 23-fold the normal expression levels in Group 6, 24 hours after LPS-treatment (Figure 2B). The PCR products of NGAL were confirmed by 2 % agarose gel electrophoresis imprinting (Figure 2C). These results are similar to previously published data from a patient study [22].

### Urinary NGAL levels corresponded to NGAL mRNA expression

**Figure 3:**
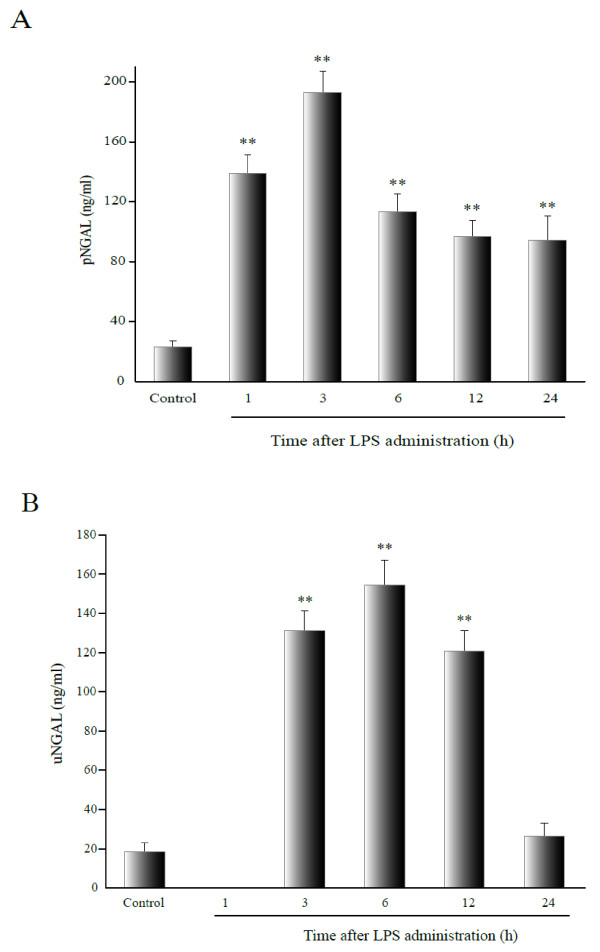
**Plasma and urinary NGAL levels in the rats subjected to LPS-treatment.** Data represent the mean ± SEM of five experiments. Abbreviations: pNGAL = plasma NGAL; uNGAL = urinary NGAL. **P < 0.001, relative to the control group.

Plasma and urinary NGAL levels increased soon after LPS administration. Compared with the control group, pNGAL levels were markedly increased in experimental groups and still persisted at high levels in Group 6 (24 hours after administration) (*P* < 0.001), by which time the renal damage had disappeared. Peak pNGAL levels (192.68 ± 14.37 ng/ml) were obtained by 3-hour post-LPS treatment (Group 3, Figure 3A).

With regard to uNGAL, the LPS-treated rats showed anuria at the early stages following LPS-treatment. However, uNGAL levels increased from 3 to 12 hours (Groups 3, 4 and 5, *P* < 0.001), and they then decreased to normal levels in Group 6, 24 hours after LPS-treatment (*P* = 0.194). Peak uNGAL values (154.42 ± 12.75 ng/ml) were observed at the 6-hour time point (Group 4). The trends observed for uNGAL levels were similar to the results of NGAL qRT-PCR analysis, and there was a correlation between the two (r = 0.850, *P* < 0.001). The correlation between pNGAL and NGAL mRNA in the injured kidney was not significant (Table 2, Figure 3B).

**Table 2:** Correlation between renal inflammatory factors, humoral NGAL levels and NGAL mRNA in LPS-induced AKI rats

	NGAL mRNA	
	r	*p*
TNFα mRNA	0.995	*P* < 0.001
IL-6 mRNA	0.276	*P* = 0.653
uNGAL	0.850	*P* < 0.001
pNGAL	0.328	*P* >0.05

Abbreviations: NGAL, neutrophil gelatinase associated lipocalin; TNFα, tumor necrosis factor α; IL-6, interleukin 6; uNGAL, urinary NGAL; pNGAL, plasma NGAL.

### TNFα and NGAL mRNA are strongly correlated

**Figure 4:**
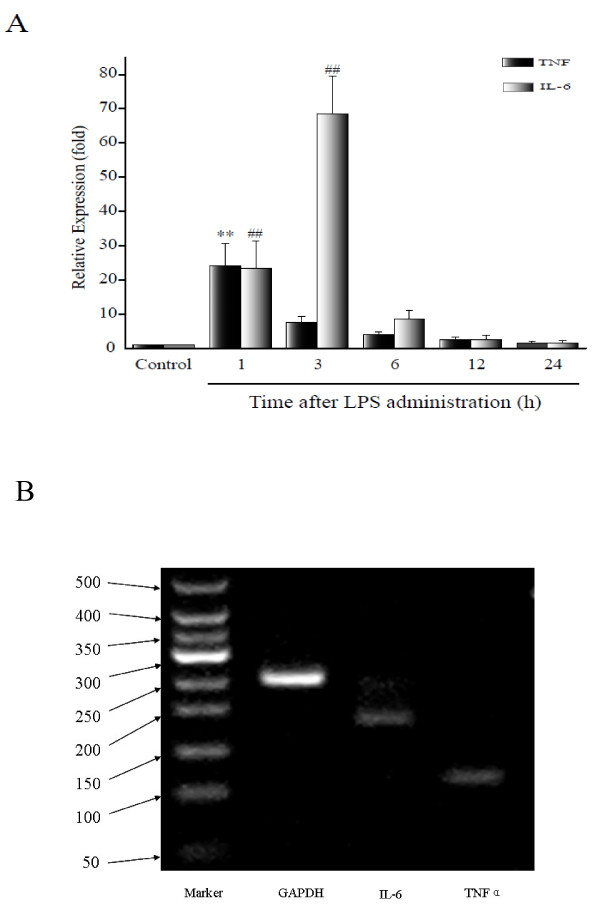
**Expression of TNFα mRNA and IL-6 mRNA in the LPS-treated rats.A**, Relative expression of TNFα and IL-6 mRNA. LPS treatment induced upregulation of TNFα and IL-6 mRNA levels, increasing the expression of both mRNAs by 24-fold at 1 hour following LPS-treatment, and IL-6 expression rose by 68-fold at 3 hours post-treatment. **B**, Agarose gel electrophoresis imprinting for TNFα and IL-6 mRNA at 1 hour after LPS administration. **P < 0.001, relative to the TNFα control group; ^##^*P* < 0.001, relative to the IL-6 control group.

There was an obvious inflammatory reaction in the kidneys following LPS treatment. Both TNFα and IL-6 mRNA expression were significantly increased at the early stage of LPS-induced AKI (*P* < 0.001). Specifically, TNFα mRNA increased by 24-fold in the first hour (Group 2) and then decreased to baseline levels 3 hours after treatment (Group 4). Similarly, expression of IL-6 mRNA also increased 24-fold in the first hour (Group 2), but the expression of IL-6 continued to increase to 68-fold greater than baseline levels at 3 hours post-treatment (Group 3), and then decreased to baseline levels at 6 hours (Group 4). There was a strong correlation between TNFα and NGAL mRNAs (r = 0.995, P < 0.001), but IL-6 mRNA was not correlated with NGAL mRNA (Table 2, Figure 4).

## Discussion

Almost half of all septic patients develop AKI [23]. Despite advances in treatment strategies for these patients, mortality due to septic AKI in critically ill patients remains high, with a mortality rate up to 60 % [3]. Of those patients that survive, 5-20 % will require dialysis [24]. The poor clinical outcome of septic AKI patients partly reflects the lack of knowledge regarding the pathophysiology of septic AKI. In addition, early diagnosis of septic AKI with markers of renal function, such as serum creatinine, which would allow for initiation treatment regimens before the disease process before becoming irreversible, are currently inadequate. In response to these difficulties, there has been a concerted effort to use high-throughput technologies such as proteomics to identify novel early biomarkers of AKI. The identification and study of NGAL marks an important step forward to identify these biomarkers [25,26]. Human NGAL was originally identified in neutrophils as a 25 kDa protein covalently bound to matrix metalloproteinase-9 (MMP-9) [27]. Importantly, NGAL fulfills many of the characteristics of a suitable “real-time” renal biomarker. For instance, it has been suggested that serum/urine NGAL levels, in addition to the intensity of NGAL staining, may serve as an early biomarker for renal injury following kidney transplantation [28-31]. Further, NGAL gene is also significantly up-regulated in the kidney following an ischemic insult [5] and has been demonstrated to be a highly predictive biomarker of other acute and chronic kidney injuries [32-36].

There are several studies that have analyzed the diagnostic and predictive properties of serum, plasma and urine NGAL in newborn [37] and adult patients critically ill with septic AKI [10,38,39]. Despite the interest in NGAL, particularly as a biomarker, there is much uncertainty and divergence regarding its use within the literature. There are several possible explanations for these differences. First, NGAL is a member of the lipocalin superfamily. This is consistent with the role of NGAL as an endogenous bacteriostatic protein that scavenges bacterial siderophores [40]. Therefore, NGAL is significantly increased in bacterial infections compared with viral infections [41,42]. Furthermore, increased sNGAL levels have been reported during systemic disease in the absence of overt bacterial infection, most notably during the acute-phase response [43], inflammation [10,44-46], hypertension [47] and chronic disease [48,49], as well as during renal tubular injury. It should be noted that patients with sepsis typically present with co-morbidities. Because NGAL levels may be influenced by these other conditions, the adoption of NGAL as a biomarker of septic AKI must be tempered with caution.

There is limited knowledge of the dynamic changes in NGAL expression during renal injury, especially in the case of sepsis-induced renal injury. Therefore, there are many questions that still need to be answered. One question is “Where is the cellular source of NGAL in the damaged kidney”? In a study by Mori et al. [22], weak NGAL staining was observed in approximately 10 % of the total cortical area of the distal tubules, and in the medullary collecting ducts of the normal kidneys from healthy volunteers. In contrast, the proximal tubules did not express NGAL. However, in ischemic or nephrotoxin-damaged kidneys from patients, nearly 50 % of the cortical tubules, including the proximal tubules, expressed NGAL. In a mouse models of renal ischemia-reperfusion injury, Mishra and colleagues verified that the NGAL protein is expressed predominantly in the proliferating cell nuclear antigen-positive proximal tubule cells within 3 hours of ischemia [5]. For the first time, we detected NGAL gene expression in 24.3-49.6 % cortical tubular epithelia, including proximal epithelia, from 1 to 12 hours following LPS administration, which is similar to the findings by Mori and Mishra. However, in the study of Paragas et al. [50], they found that NGAL mRNA was not expressed by epithelia in proximal tubules in the rat at 24 hours after treatment with lipid A (15 mg/kg, i.p.), but the medullary cells demonstrated intensive NGAL mRNA expression. These data described above suggest the following. 1) Different parts of the kidney have different responses to factors causing injury, including endotoxin, but overall, the injured epithelia are the source of NGAL. 2) Epithelia in proximal tubules might be sensitive to endotoxin, and they can upregulate the NGAL gene even though the LPS concentration is low, but the reactive period is short. Thus, NGAL expression can be detected in the early stage after LPS administration, and then decrease to baseline levels. 4) The cells in the medullary tubules might be insensitive, and they will not become active until they face a high concentration of LPS. However, they can increase NGAL expression longer than the cells in proximal tubules.

Moreover, the relationship between NGAL in renal injury and humoral NGAL needs to be clarified. Paragas and colleagues [45] verified that uNGAL originates in the kidney from results of cross-transplants between NGAL knockout and wild-type mice, followed by renal artery clamping. They found that NGAL is present in the kidney, liver, spleen, lung and trachea after lipid A treatment, which indicates that NGAL in the blood is not a good marker of septic AKI [50]. Our study revealed, for the first time, the pattern of renal NGAL expression during the early stage of endotoxemia. We found that renal NGAL could be a useful biomarker of renal epithelia injury. In particular, uNGAL exactly reflected the change in renal NGAL expression, whereas pNGAL was not as accurate in septic AKI. Therefore, based on the findings of these studies, it appears that uNGAL levels are related to NGAL gene expression in the kidney, and uNGAL has the ability to act as a marker for the diagnosis and monitoring of AKI in patients with sepsis. This could lead to renoprotective therapies and avoidance of renal injury. However, pNGAL or sNGAL levels may be misleading in the diagnosis of septic AKI. Misdiagnosis may result in conservative strategies and the optimal therapeutic time in critically ill patients may be missed.

Sepsis is associated with the production of many inflammatory mediators, including TNFα [51,52]. TNFα is released first when sepsis occurs and leads to cleavage of the nuclear factor κB (NF-κB) inhibitor. Once this inhibitor is removed, NF-κB is able to initiate the production of mRNA, which induces the production of other proinflammatory cytokines, chemokines, and adhesion molecules [53]. For instance, Knotek et al. demonstrated that TNFα is a critical mediator of endotoxin-induced sepsis, and that TNFα inhibition prevents physiological changes and morbidities associated with LPS administration in wild-type and inducible nitric oxide synthase knockout mice [54]. Therefore, TNFα is a key mediator of LPS-induced acute renal failure, acting through its receptor, TNFR1 [55]. Early changes in renal TNFα mRNA levels after endotoxemia have not been reported. In our study, we found that TNFα mRNA was induced by 24-fold within 3 hours after LPS treatment. The result of our study also confirm the findings of Wang and colleagues, they showed that LPS induced TNF-α protein by 24-fold in the kidney 16 hours after administration[14]. Furthermore, for the first time, we showed that NGAL mRNA was related to TNFα mRNA levels in the injured kidney, and that NGAL mRNA upregulation closely followed TNFα mRNA increase, similar to a down-regulation cytokine in the inflammation cascade. Previous studies [22,50] have found that NGAL is suppressed by the NF-κB inhibitor in primary kidney cells after lipid A administration, and that the NGAL:siderophore:Fe complex preserves proximal tubule N-cadherin and inhibits cell death. Therefore, is it possible that TNFα/NF-κB/NGAL is an important mechanism for regulating tubular cells by increasing proliferation and/or inducing/suppressing apoptosis during sepsis? This possibility should be investigated in future studies.

## Conclusions

The upregulation of NGAL gene expression in the tubular epithelia occurs after LPS-treatment, and this finding is coupled with the observation of renal TNFα increase and injury. Urinary NGAL levels accurately reflect the changes in NGAL mRNA in this kidney injury model.

## Competing interests

The authors have no competing interests to declare.

## Authors’ contributions

MH collected samples, performed the histology and in situ hybridization analysis, performed the qRT-PCR and ELISA, and wrote and edited the manuscript. YL coordinated the in vivo studies and edited the manuscript. MDL coordinated the in vitro studies and edited the manuscript. YML and BC edited the manuscript. All authors read and approved the final manuscript.

## Pre-publication history

The pre-publication history for this paper can be accessed here:

http://www.biomedcentral.com/1471-2369/13/25/prepub
